# Robust and Unbiased Variance of GLM Coefficients for Misspecified Autocorrelation and Hemodynamic Response Models in fMRI

**DOI:** 10.1155/2009/723912

**Published:** 2009-09-06

**Authors:** Lourens Waldorp

**Affiliations:** University of Amsterdam, Roetersstraat 15, 1018 WB Amsterdam, The Netherlands

## Abstract

As a consequence of misspecification of the hemodynamic response and noise variance models, tests on general linear model coe cients are not valid. Robust estimation of the variance of the general linear model (GLM) coecients in fMRI time series is therefore essential. In this paper an alternative method to estimate the variance of the GLM coe cients accurately is suggested and compared to other methods. The alternative, referred to as the sandwich, is based primarily on the fact that the time series are obtained from multiple exchangeable stimulus presentations. The analytic results show that the sandwich is unbiased. Using this result, it is possible to obtain an exact statistic which keeps the 5% false positive rate. Extensive Monte Carlo simulations show that the sandwich is robust against misspeci cation of the autocorrelations and of the hemodynamic response model. The sandwich is seen to be in many circumstances robust, computationally efficient, and flexible with respect to correlation structures across the brain. In contrast, the smoothing approach can be robust to a certain extent but only with specific knowledge of the circumstances for the smoothing parameter.

## 1. Introduction

Brain activity maps from functional magnetic resonance imaging (fMRI) time series are becoming increasingly important in the cognitive sciences [1]. An fMRI brain activity map contains thousands of volume elements (voxels) that make up the entire brain. For each of these voxels a blood-oxygenation level dependent (BOLD) time series is available. In order to increase the signal-to-noise ratio, exchangeable stimuli are repeated several times in experiments [2]. Since there are many voxels, analyses are often performed voxelwise to decrease computational load (mass univariate approach). In the general linear model (GLM), the time series of each voxel is represented by a linear combination of modeled time series corresponding to a condition or effect [3]. Amplitude coefficients and their variances are then computed such that hypothesis testing can be performed on (a function of) these coefficients to, for example, test between conditions. This paper is about estimating the variance of the amplitude coefficients as accurately as possible such that hypothesis testing is valid.

Hypothesis tests on functions of parameters are greatly influenced by the estimate of the variance of the model parameters, which in turn is greatly influenced by the autocorrelations of the time series [1, 4, 5]. Generally, two approaches to estimate the variance of the coefficients can be distinguished: (i) transforming the data such that the time series becomes uncorrelated or “white,” and (ii) transforming the data such that the data are smoothed or “colored,” and then using the known, smooth structure for variance estimation [6, 7]. In prewhitening, on the one hand, a model for the autocorrelations of the time series is used which should render the data uncorrelated [8]. Often an autoregressive (AR) process is used [9], but many other strategies exist [10–13]. The advantage of prewhitening is that the obtained variance estimate is the smallest compared to all other unbiased estimates [14]. However, this advantage holds only if the model for the correlation structure is correct [7], which is, of course, difficult to maintain. It has been suggested that accounting for bias due to autocorrelations is not required because the estimates did not improve enough [7]. However, Marchini and Smith [7] did not consider an incorrect correlation structure, only bias due to limited length of the time series. Precoloring, on the other hand, has the advantage that the assumed correlation structure need not be correct [4]. A disadvantage is that a smoothing parameter of, for example, a Gaussian kernel needs to be chosen (see, e.g., [15]). Such a decision can influence the quality of the variance estimate [7, 13]. Another disadvantage of the smoothing approach is that high-frequency components in the data can be attenuated [11].

In addition to misspecification of the autocorrelations, the model for the hemodynamic response is also likely to be incorrect [16]. This means that the residuals contain misspecification which is carried into the estimator of the variance of the GLM coefficients. It is therefore important to take such misspecification into account in any statistical analysis of fMRI time series. Recognizing that any model is strictly incorrect, it makes sense to consider the degree of misspecification; that is, the difference between the truth and its approximation is important instead of the actual model used.

I agree with Friston et al. [4] that robust variance takes priority over efficient variance, regardless of whether the model for the correlations is correct or not. However, optimally a robust variance estimate should also be able to adapt to local variations of correlation structure. Variation of correlation structure exists across different locations of the brain [9]. A variance estimate like the smoothing approach that works well on average of brain locations can therefore be improved. I suggest a robust variance estimate based on the residuals but taking into account the individual replications or events. This variance estimate adapts to correlational changes, is computationally efficient, and is robust. I show that this robust variance estimate is unbiased and as a result can be used for hypothesis testing even with few replications.

The paper is organized as follows. Section 2 introduces the differences between the true underlying process and the GLM, the working model. This section also discusses existing methods of estimating the variance of the coefficients and introduces the new, robust variance estimate. Subsequently, hypothesis testing is discussed for the different estimators. In Section 3 extensive Monte Carlo simulations are discussed to show how the different estimators perform in different circumstances for blocked and event-related designs.

## 2. Model Specification and Misspecification

In model specification a data generating process (DGP) is assumed to exist. This DGP is in general unknown and is therefore approximated by a working model. Such an approximation can be misspecified in at least two ways: (i) the model for the mean can be incorrect, and (ii) the model for the autocorrelations noise can be incorrect. An example of a misspecified model for the mean is using a gamma function as a model for the hemodynamic response when the BOLD response is in fact generated by the balloon model; see, for example, [16]. An example of misspecification of the autocorrelations is using an autoregressive model for temporal correlations, when the correlations are actually 1/frequency [1]. First, statistical assumptions of the DGP are described followed by misspecification of the GLM for fMRI data as a working model.

Data of *i* = 1,…, *p* time points or scans are available measured on *j* = 1,…, *n* independent trials or replications. The data are collected in the *p*-vector *Y*_*j*_. The DGP for *Y*_*j*_ is *Y*_*j*_ = *g*_*θ*_(*Z*) + *e*_*j*_, where *g*_*θ*_(*Z*) < *∞* is an unknown (non)linear, nonrandom function with fixed regressors *Z* = (*z*_1_,…, *z*_*m*_) and unknown parameters *θ*. The noise *e*_*j*_ has joint distribution function *F*(*e*) with mean zero and unknown variance *E*{*e*_*j*_ *e*_*k*_′} = Σ for *j* = *k* and zero otherwise. So, there is autocorrelation, but no correlations among replications.

The working model specifies an approximation to the DGP for the mean and the variance of the data. In the GLM a linear function *Xβ* is used as an approximation to the mean *E*{*Y*_*j*_} = *g*_*θ*_(*Z*), where *X* is a *p* × *k* matrix and *β* a *k*-vector of coefficients. The noise is assumed to have temporal correlations but remains unspecified for the moment. Then the working model on replication *j* is *Y*_*j*_ = *Xβ* + *r*_*j*_, where the residual *r*_*j*_ = *g*_*θ*_(*Z*) − *Xβ* + *e*_*j*_ contains both the modeling error *g*_*θ*_(*Z*) − *Xβ* and noise *e*_*j*_. The variance of the residual *r*_*j*_ is again Σ since the modeling error is fixed (but see below for the estimated residual). The model *Xβ* could correspond to the mean of the DGP, that is, *g*_*θ*_(*Z*) = *Xβ*, but in general they are different. It is assumed that the matrix *X* has full column rank, *r*(*X*) = *k*, such that *X*′*X* is nonsingular.

**Figure 1:**
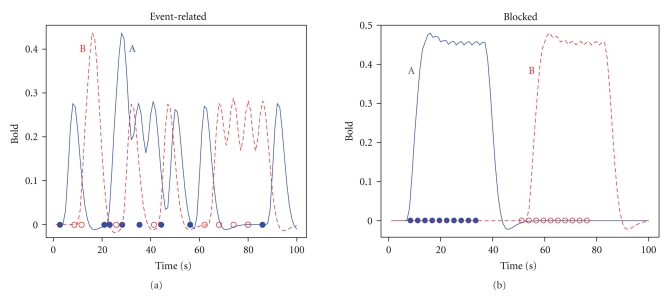
Convolution of the HRF and the stimulus function for an event-related (a) and a blocked design (b). Stimulus presentation latencies for condition A (solid blue) are indicated with filled circles, and open circles for condition B (dashed red). Parameters of the HRF are taken from [18].

The main parameters of interest in fMRI are the amplitude parameters *β* of the BOLD response time series. To model the delayed response, a hemodynamic response function (HRF) is used, convolved with the stimulus presentation timing of the experiment. A possible HRF used in analyses is a double gamma function [17, 18]. The stimulus (“on-off”) function is given by *s*(*t*) = 1 for all time points *t* that the stimulus is present and zero otherwise. An example of the convolution of the time series is given in Figure 1, where conditions A and B are the same except for presentation times. The experiment can either be event related or blocked [1, 19]. In an event-related design each presentation in a sequence can belong to any of the conditions, whereas in a blocked design a sequence of presentations for a particular condition is given in blocks (see, e.g., [1, 20]). An example of each is given in Figure 1. The convolutions form the columns of the design matrix *X*. The design matrix *X* can also include temporal derivatives to account for latencies in the BOLD signal [21, 22].

When the coefficients are estimated, a function of the estimate $\hat{\beta }$ is usually tested, which is called a contrast. The variance of a contrast $c^{\prime }\hat{\beta }$ is then $c^{\prime }\text{var}\{\hat{\beta }\}c$. A possible test of the contrast is the *F*-test

(1) $$\begin{array}{c} F=k_{n}\frac{{\left(c^{\prime }\hat{\beta }-a\right)}^{2}}{c^{\prime }\text{var}\left\{\hat{\beta }\right\}c}, \end{array}$$

where *k*_*n*_ is a factor to obtain the correct null distribution for the hypothesis $c^{\prime }\hat{\beta }=a$ [18]. This statistic is approximately *F* distributed with degrees of freedom dependent on the estimate of the contrast variance. It is clear from the definition that the statistic, and therefore the false positive rate, is directly influenced by the contrast variance. This paper is about finding a robust estimate of this contrast variance such that inference concerning *β* through hypothesis testing is valid.

### 2.1. Estimation

A general way of estimating the coefficients and their variance is explained, after which four different methods of defining an estimator are discussed. This follows mostly the presentations of [7, 12]. The four methods are also summarized in Table 1.

Let *S* be a nonsingular *p* × *p* matrix and premultiply the data, model, and residual with *S* such that *SY*_*j*_ = *SXβ* + *Sr*_*j*_. Then the variance of the residual *r*_*j*_ is *S*Σ*S*′. The least squares estimate is $\hat{\beta }=(X^{\prime }S^{\prime }SX{)}^{-1}X^{\prime }S^{\prime }S\overset{̅}{Y}$, where $\overset{̅}{Y}=\left(1/n\right)\sum_{j=1}^{n}Y_{j}$. Because the HRF model is misspecified, $\hat{\beta }$ is biased, that is,

(2) $$\begin{array}{c} E\left\{\hat{\beta }\right\}={\left(X^{\prime }S^{\prime }SX\right)}^{-1}X^{\prime }S^{\prime }Sg_{\theta }\left(Z\right)=\beta^{*}. \end{array}$$

The mean β* can be described as a least squares approximation to the unknown function *g*_*θ*_(*Z*), which is very different from linearization of *g*_*θ*_(*Z*) in terms of a first-order Taylor expansion. The main difference between the least squares and Taylor approximation is that the first describes the nonlinear function on the whole range of *Z*, whereas the latter is accurate only in a neighborhood of a specific *Z* (see [23] for more details on this). The variance of $\hat{\beta }$ is

(3) $$\begin{array}{c} \text{var}\left\{\hat{\beta }\right\}=\frac{1}{n}{\left(X^{\prime }S^{\prime }SX\right)}^{-1}X^{\prime }S^{\prime }S\Sigma S^{\prime }SX{\left(X^{\prime }S^{\prime }SX\right)}^{-1}. \end{array}$$

Given $\hat{\beta }$, an estimate of the residual is given by

(4) $$\begin{array}{c} {\hat{r}}_{j}=\left(I_{p}-H_{SX}\right)Sg_{\theta }\left(Z\right)-H_{SX}S\overset{̅}{e}+Se_{j}, \end{array}$$

where *H*_*SX*_ = *SX*(*X*′*S*′*SX*)^−1^ *X*′*S*′ and $\overset{̅}{e}=\left(1/n\right)\sum_{j=1}^{n}e_{j}$. The mean and variance of the estimated residual are

(5) $$\begin{array}{c} E\left\{{\hat{r}}_{j}\right\}=Q_{SX}Sg_{\theta }\left(Z\right),\\ \text{var}\left\{{\hat{r}}_{j}\right\}=\frac{1}{n}Q_{SX}S\Sigma S^{\prime }Q_{SX}+\frac{n-1}{n}S\Sigma S^{\prime }, \end{array}$$

where *Q*_*SX*_ = *I*_*p*_ − *H*_*SX*_. These results are different from other derivations in three ways (see, e.g., [6, 7]): (i) the estimator $\hat{\beta }$ is biased because the incorrect model is used for the mean, (ii) the expectation of the estimated residual is not zero because $\hat{\beta }$ is biased, and (iii) the variance of the estimated residual ${\hat{r}}_{j}$ contains two terms, one with the design matrix *X* and one without *X*, because the number of replications is taken into account. Especially this last point will be used to our advantage, as described below.

The objective is to obtain an unbiased estimate of $\text{var}\{\hat{\beta }\}$ without any additional modeling of the autocorrelations. So, we set *S* = *I*_*p*_ and obtain the so-called ordinary least squares estimate ${\hat{\beta }}_{O}$. If we plug in (5) into (3), we see that only the second part containing Σ will remain because *X*′*Q*_*X*_ = 0. So, we need an estimate of $\text{var}\{{\hat{r}}_{j}\}$ to make this work. Suppose that we use

(6) $$\begin{array}{c} W=\frac{1}{n-1}\overset{n}{\underset{j=1}{\sum }}{\hat{r}}_{j}{\hat{r}}_{j}^{\prime }. \end{array}$$

From the variance of the residual in (5) it can be seen that for the expectation of *W* we have

(7) $$\begin{array}{ll} E\left\{W\right\} & =\frac{1}{n-1}\overset{n}{\underset{j=1}{\sum }}E\left\{{\hat{r}}_{j}{\hat{r}}_{j}^{\prime }\right\}\\ & =\frac{n}{n-1}Q_{X}\left(g_{\theta }\left(Z\right)g_{\theta }{\left(Z\right)}^{\prime }+\frac{1}{n}\Sigma \right)Q_{X}+\Sigma . \end{array}$$

Then we have for the variance of ${\hat{\beta }}_{O}$

(8) $$\begin{array}{ll} E\left\{{\hat{V}}_{W}\right\} & =\frac{1}{n}{\left(X^{\prime }X\right)}^{-1}X^{\prime }E\left\{W\right\}X{\left(X^{\prime }X\right)}^{-1}\\ & =\frac{1}{n}{\left(X^{\prime }X\right)}^{-1}X^{\prime }\Sigma X{\left(X^{\prime }X\right)}^{-1}, \end{array}$$

as required. It works because of the two-part variance in (5), and there are two parts in the variance because we took into account the number of replications obtained in the experiment. This estimator is for obvious reasons sometimes referred to as the sandwich estimator [24]. In general the sandwich can be shown to be consistent; that is, the estimator will be correct for large *n* [23]. In this particular case where the design matrix is fixed, the sandwich estimator is even unbiased, which is usually not the case. As a consequence, the sandwich is accurate for few number of replications *n*. The fact that the sandwich is unbiased without any specification of smoothing or a model for the noise correlation structure is especially appealing. Another advantage is that because the residuals are used, the sandwich estimator adapts itself according to the correlation structure of each voxel. So, it is flexible, computationally efficient, and robust. These facts of the sandwich can be used to create an exact test, shown in the next section.

Three other common estimators of $\text{var}\{\hat{\beta }\}$ will be discussed briefly for comparison. The simplest one is ordinary least squares (OLS). It is obtained by assuming that the noise variance is Σ = *σ* ^2^ *I*_*p*_ and setting *S* = *I*_*p*_. Then the variance of the OLS estimate ${\hat{\beta }}_{O}$ is obtained by estimating the scalar noise variance *σ* ^2^, which is estimated by the sum of the squared residuals [1]. The OLS estimator of the variance of ${\hat{\beta }}_{O}$ is then ${\hat{V}}_{O}={\hat{\sigma }}_{O}^{2}(X^{\prime }X{)}^{-1}$. This estimator is biased because the estimator ${\hat{\beta }}_{O}$ is biased because from (4) we have $E\{{\hat{r}}_{j}^{\prime }{\hat{r}}_{j}\}=g_{\theta }{\left(Z\right)}^{\prime }Q_{X}g_{\theta }(Z)+\sigma^{2}$. It is well known that if there are autocorrelations, then OLS will lead to variance estimates that are too small (see also simulation section below); see for example, [4, 25, 26].

The second estimator is called (feasible) generalized least squares (GLS). It is obtained by assuming that there are autocorrelations and these are estimated. Then set *S* such that the estimate of the noise variance is $\hat{\Sigma }=SS^{\prime }$ [8]. The variance of the GLS coefficient ${\hat{\beta }}_{G}$ is often written as a product of a scalar variance and a correlation matrix, Σ = *σ* ^2^ *R*. Then the estimate of *σ* ^2^ using ${\hat{\beta }}_{G}$ in the residuals is obtained similarly to OLS and is referred to as ${\hat{\sigma }}_{G}^{2}$. The correlation matrix *R* can be estimated by any number of suggested algorithms. Often an AR(*p*) process is assumed for *R* with *p* = 1, 2 [9, 18], or sometimes higher [27]. Other GLS methods include transforming the time series to the frequency domain [10–12] and transforming the time series to the wavelet domain, retaining the correlation structure to obtain an estimator for *R* [13]. The variance of the coefficient ${\hat{\beta }}_{G}$ estimated by GLS is ${\hat{V}}_{G}={\hat{\sigma }}_{G}^{2}(X^{\prime }{\hat{R}}^{-1}X{)}^{-1}$. It is known that if the model for the variance is correct, then GLS is most efficient; that is, the estimator attains the Cramér-Rao lower bound of the variance of all unbiased estimates [14]. The problem is that it is very difficult to find an unbiased estimate of *R*, even for large time series (large *p*, note the difference in asymptotics with the sandwich), not in the least because the model used for the temporal correlations is incorrect [4, 28, 29]. If no correct model is known, then GLS could lead to very inaccurate variance estimates for the coefficients *β*. Friston et al. [4] show clearly that assuming an incorrect model for the noise correlations can lead to variance estimates that are too high or too low (see also the section Monte Carlo Simulations).

The third estimator is called the smoothing approach, sometimes called precoloring. It is obtained by assuming that Σ = *σ* ^2^ *R*, with *R* a correlation matrix, and setting *S* such that $SRS^{\prime }\approx S\hat{R}S^{\prime }$ [30]. So, the temporal correlations in the time series are dominated by a smoothing matrix *S* such that the true temporal correlations become irrelevant to estimating the variance of the coefficient ${\hat{\beta }}_{S}$. Then *σ* ^2^ is estimated by ${\hat{\sigma }}_{S}^{2}$, which is the average squared residuals divided by the degrees of freedom [30]. The estimator ${\hat{\sigma }}_{S}^{2}$ is biased if ${\hat{\beta }}_{S}$ is biased. The correlation matrix *R* can be estimated, which can be done in the same manner as described above for GLS, for example with an AR(*p*) model [18]. The variance estimator for the coefficient ${\hat{\beta }}_{S}$ using a smoothing matrix *S* is (3) with $\hat{\Sigma }={\hat{\sigma }}_{S}^{2}\hat{R}$, which is referred to as ${\hat{V}}_{S}$. The smoothing matrix is often generated by the Gaussian function exp[−(*i* − *j*)^2^/2*τ* ^2^], where *i* is the row, *j* is the column of *SS*′, and *τ* ^2^ is the variance [31]. Suggested values for *τ* ^2^ are 4 to 8 s^2^. An advantage of ${\hat{V}}_{S}$ is that it is robust against using an incorrect model for *R*, which is likely to be the case. However, it is in general difficult to set *S* such that $SRS^{\prime }\approx S\hat{R}S^{\prime }$ for each correlation structure [7]. Friston et al. [4] suggest a bandpass filter for *S* which minimizes the the squared difference for a contrast between the true and estimated variance over all possible (autoregressive) correlations in the time series. This will result on average in a reasonable estimate for all voxels with different correlation strengths which is computationally efficient. Optimally, however, one would like to use the same estimator for each voxel that somehow adapts to the particular correlation strengths of that voxel.

### 2.2. Hypothesis Testing

Contrasts are used to create a function of the coefficient that will allow to test for differences between conditions. For example, a single contrast could be *c*′ = (1, −1), to test between the amplitudes of different conditions. An *F*-test can be used to test the null hypothesis *H*_0_ : *c*′*β* = *a* against the alternative *H*_*A*_ : *c*′*β* ≠ *a*. Depending on which estimator for *β* and which variance estimate is used, a specific *F*-test will result. For the simple contrast like *c*′ = (1, −1) and *a* = 0 the *F*-test is the square of the *t*-test. In general, for a set of *q* independent contrasts, collected in the *q* × *k* matrix *C*, the *F*-test is [32]

(9) $$\begin{array}{c} F=\frac{n-q}{nq}{\left(C\hat{\beta }-a\right)}^{\prime }{\left(C\hat{V}C^{\prime }\right)}^{-1}\left(C\hat{\beta }-a\right), \end{array}$$

which under *H*_0_ is distributed approximately as *F* with degrees of freedom dependent on the statistic for the variance $\hat{V}$ (see Table 1). If OLS or GLS is used, then the statistics *F*_*O*_ and *F*_*G*_ are approximately *F*(*q*, *p* − *k*) distributed. If the smoothing approach is used, then usually the so-called Satterthwaite approximation *f*_*S*_ to the degrees of freedom is used, which depends on both the autocorrelation and the design [7, 30]. So, for the smoothing approach, the statistic *F*_*S*_ is approximately *F*(*q*, *f*_*S*_) distributed. Finally, if the sandwich estimator is used, an exact test *F*_*W*_ exists which is *F*(*q*, *n* − *q*) distributed, provided that the data are multivariate normal, that is, if *F*(*e*) = *N*_*p*_(0, Σ) (see appendix for details on this). The degrees of freedom do not contain the length of the time series (*p*) because the correlation structure of the time series is entirely estimated from the information of the replications. The fact that it is an exact test means that even for very small number of replications *n* the *F* statistic is very accurate, that is, has a false positive rate of 5%, say. The assumption of multivariate normal noise in fMRI is important, of course, and has been investigated. It appears that the assumption of Gaussian noise is valid in general for low and high signal-to-noise ratios and is very accurate when considering difference images, as is often the case in fMRI analyses [33].

## 3. Monte Carlo Simulations

In this section Monte Carlo simulations are used to show in which circumstances each of the four variance estimates works best. This is done by considering four variables: (i) the autocorrelation of the time series, (ii) misspecification of the correlation structure, (iii) misspecification of the mean model, and (iv) the type of design. The focus of these simulations is on model misspecification instead of specific models for the HRF and autocorrelations. In so doing the results of these simulations apply to many different situations with different models but similar misspecification.

### 3.1. Data Generation

A time series is created of fMRI data of length *p* = 100 seconds. The data generating process is linear in the parameters, *g*_*θ*_(*Z*) = *Zθ*. The columns of the design matrix *Z* = (*z*_1_, *z*_2_) are generated according to the double gamma function and represent time series corresponding to two different experimental conditions A and B of either an event-related or a blocked design [3]. The event-related design was generated using random stimulus presentations with 8 presentations per condition in the 100 second interval with the constraint that the interstimulus interval was at least 2 seconds. In the blocked design there was one block for each of the two conditions with 10 stimulus presentations in each block. The exact designs used are shown in Figure 1. The parameter *θ* represents the amplitude of the BOLD response corresponding to a condition. Noise *e*_*j*_ is added to the signal *Zθ* which is *N*_*p*_(0, Σ) for *j* = 1,…, *n* with Σ = *σ* ^2^ *R*. The correlation matrix *R* = (*ρ*)_*ij*_ is induced by either an AR(1) or AR(2) process, which are, respectively, *U*(*t*) = *ϕU*(*t* − 1) + *ε*(*t*) and *U*(*t*) = *γ*_1_ *U*(*t* − 1) + *γ*_2_ *U*(*t* − 2) + *ε*(*t*), where *ε*(*t*) is white noise [35]. The coefficients of the AR(2) process have been sampled from the upper right quadrant of the stationary area: 0 < *γ*_1_ + *γ*_2_ < 1 [35]. A single parameter is created to indicate strength of dependence in the time series *ϕ* = *γ*_1_ + *γ*_2_, which is varied from 0.2 to 0.9, with *γ*_1_ at most 0.1 larger than *γ*_2_. This also reflects the possible differences in correlation structure as found between voxels. The variance of the time series at *t* = 0 is taken as *σ*_0_^2^ = 1. Then the data are *Y*_*j*_ = *Zθ* + *e*_*j*_ for *j* = 1,…, *n*. The variance of the noise is set such that the signal-to-noise ratio (SNR) for the time series is approximately one for the average over replications. This is achieved by multiplying the variance of the noise by the number of replications. As a consequence the number of replications is irrelevant; only the SNR is important which is set at an appropriate low level (see [36]).

### 3.2. Estimation

**Figure 2:**
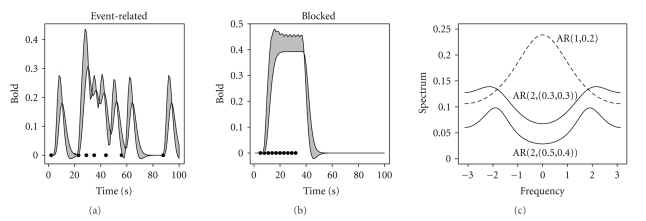
(a) and (b) Misspecification of the HRF for condition A with the largest relative difference of 0.278 for the event-related design and 0.149 for the blocked design. (c) Three spectra of AR processes are displayed as a function of frequency for [−*π*, *π*] [34]. The AR(1) process was generated with parameter *ϕ* = 0.2, and the two AR(2) processes are generated with *γ*_1_ = *γ*_2_ = 0.3, *γ*_1_ = 0.5, and *γ*_2_ = 0.4.

Estimation with the working model *Y*_*j*_ = *Xβ* + *r*_*j*_ is performed using a different HRF, *h*(*t*)*, which is a single gamma function [1]. The resulting time series form the columns of *X* in the working model, such that *Z* ≠ *X*, and as a result *θ* ≠ *β*. The main difference between the functions is that there is no undershoot using the single gamma function. Additionally, a parameter is varied in the single gamma function to vary the degree of misspecification. At the largest misspecification this induces a reduction of amplitude to about 30% and a delay of about 2 seconds, shown in Figure 2. To quantify the difference between the DGP and working model, the relative difference between the functions is computed, defined as the sum of the absolute difference between the functions divided by their sum over the whole range. This relative difference was for the event-related design between 0.072 and 0.278 and for the blocked design between 0.075 and 0.149. The lowest relative difference is solely due to selecting the incorrect single gamma function. The largest effect of misspecification is in the event-related design. This is to be expected since the shape of the HRF is more important in event-related designs [1].

The misspecification in the correlation structure for GLS and the smoothing approach is created by using as a working model an AR(1) instead of an AR(2). The amount of misspecification depends on the correlation strength of the generated structure with AR(2); see Figure 2. It is clear that estimating the correlation structure using an AR(1) process will capture mostly frequencies around zero, whereas it will represent poorly frequencies further away from zero.

**Figure 3:**
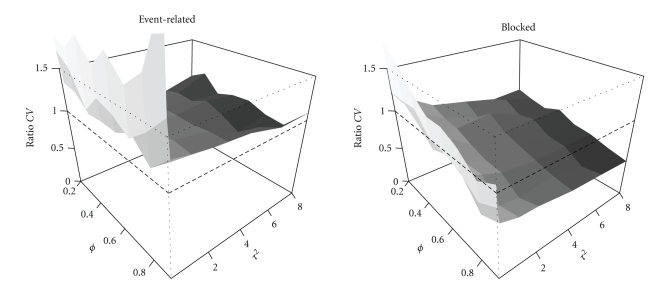
Ratios of estimated and true contrast variance for event-related and blocked designs as a function of correlation strength *ϕ* and smoothing parameter *τ* ^2^ for the smoothing approach.

The smoothing approach requires setting the smoothing matrix *S* by the parameter *τ* ^2^. The value of this parameter depends on both the correlation strength and the design. Therefore, we first looked at the effect on the variance estimate for different values of correlation strength *ϕ* and *τ* ^2^. As can be seen in Figure 3, there is no absolute correct value of *τ* ^2^ for both event-related and blocked designs and all correlation strengths when only the correlation structure is misspecified. The value of *τ* ^2^ = 8 seems to be most optimal in the sense that it is robust against correlation strength, especially in the event-related design. This value is used in the simulations for the smoothing approach unless specified otherwise. 

To compare the four approaches three measures are discussed: the ratio of estimated to true contrast variances, the false positive rate, and power. The contrast tested is *c*′ = (1, −1). The true contrast variance is obtained by computing the variance from the *N* = 500 simulations of the estimate $\hat{\beta }$ for each of the methods. Note that the true variance is defined differently from that defined in [4], where a second-order approximation to the mean squared error was used. The bias formulation ignored stochasticity of the estimated correlation matrix $\hat{R}$ which was approximated to the second order. Let *D* denote the true variance obtained from the *N* simulations. The ratio of contrast variance is then $c^{\prime }\hat{V}c/c^{\prime }Dc$. If the estimated variance is good, then the ratio will be 1, it is overestimated if the ratio is larger than 1, and it is underestimated if the ratio is smaller than 1.

The false positive rate or size of a test is the probability of a test to reject the null hypothesis when it is true. The false positive rate (FPR) is set at 5%. It is expected that when the contrast variance is underestimated, then the FPR will be too high, that is, higher than 5%, and when the contrast variance is overestimated, the FPR will be too low. In relation to FPR, power is compared between methods as a function of effect size. Power refers to the probability of rejecting the null hypothesis when it is incorrect. Power should be close to 1 given a sufficient effect size. Effect size *η* is here defined as the difference between amplitude parameters divided by the true contrast variance. If the FPR is too low, then the power will also be low, and when the FPR is too high, the power will be high.

### 3.3. Results

We first look at the contrast variance when the assumptions about the correlation structure and HRF are correct. Then we determine the effect of misspecification of the autocorrelations on the contrast variance, FPR, and power. And finally we look at possible interactions of misspecification of the autocorrelations and the HRF.

**Figure 4:**
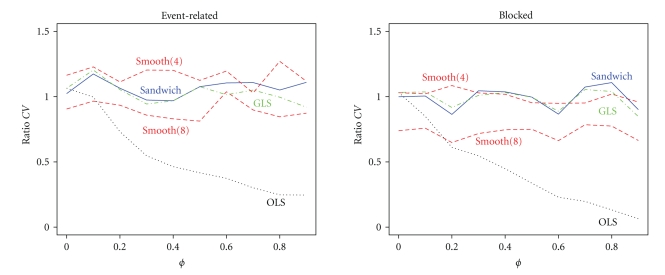
Ratios of estimated and true contrast variance when the correlation structure is correctly specified as an AR(1) process as a function of the AR(1) parameter *ϕ*. The methods displayed are: OLS (black, dotted line), GLS (green, dashed-dotted line), smooth with *τ* ^2^ = 8 (red, dashed line), smooth with *τ* ^2^ = 4 (red, long-dashed line), and sandwich (blue, solid line).

When both the HRF and autocorrelations are correctly specified, all methods should work well, except OLS when there are autocorrelations. In Figure 4 it is clearly seen that for the event-related and blocked design both the sandwich and GLS perform equally well for any value of *ϕ*. As expected, OLS is close to one only when *ϕ* = 0. In the event-related design the contrast variance of the smoothing approach with *τ* ^2^ = 8 is quite close to one, but the contrast variance for this *τ* ^2^ is underestimated in the blocked design. In the blocked design the contrast variance is very accurate for all values of *ϕ* when *τ* ^2^ = 4. So, when the model for the noise variance is correct, the sandwich is almost exactly the same as the minimum variance GLS regardless of design. The smoothing approach, on the other hand, depends strongly on the design, and different smoothing parameters are required for accurate contrast variance estimates.

**Figure 5:**
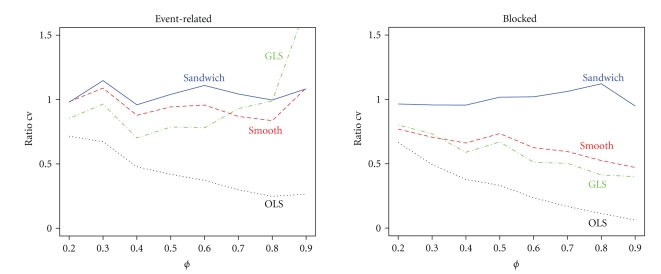
Ratios of estimated and true contrast variance when the correlation structure is misspecified for the four methods for both the event-related and blocked design as a function of correlation strength *ϕ*.

**Figure 6:**
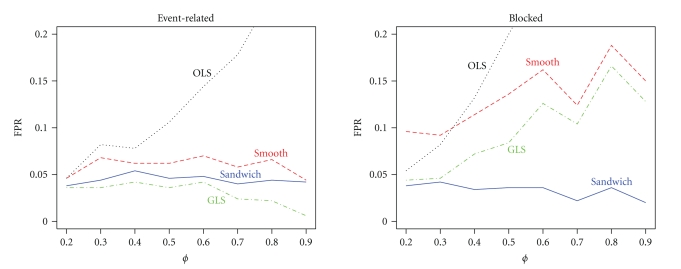
False positive rate as a function of correlation strength *ϕ* for the event-related and blocked design when the correlation structure is incorrect.

**Figure 7:**
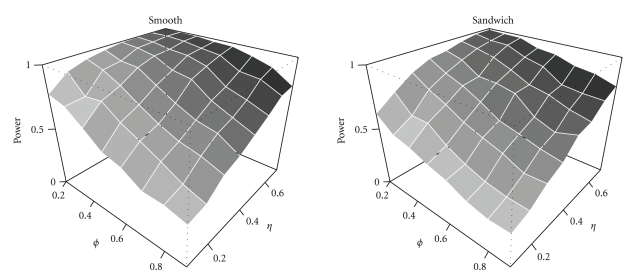
Power for the event-related design as a function of correlation strength *ϕ* and effect size *η*.

If there is misspecification in the correlation structure, then the contrast variance of a robust estimator should still be accurate for all levels of correlation strength. It is clear from Figure 5 that now OLS and GLS perform poorly. OLS always underestimates the true contrast variance, and GLS either underestimates or overestimates contrast variance. Both the smoothing approach and the sandwich are robust for misspecification of the correlation structure in the event-related design. However, in the blocked design only the sandwich is robust at all levels of correlation strength. As a consequence the smoothing approach has a slightly higher FPR than the nominal 5% in the event-related design but a dramatically higher FPR in the blocked design, shown in Figure 6. This was expected because from Figure 5 the contrast variance was underestimated, and so the FPR is expected to be too high. In contrast, the sandwich has FPR slightly below the nominal 5% in both designs because it overestimated the contrast variance slightly. In accordance with the contrast variance and FPR results, the power of the smoothing approach is slightly higher than that of the sandwich, as can be seen in Figure 7. The power for the blocked design is comparable.

**Figure 8:**
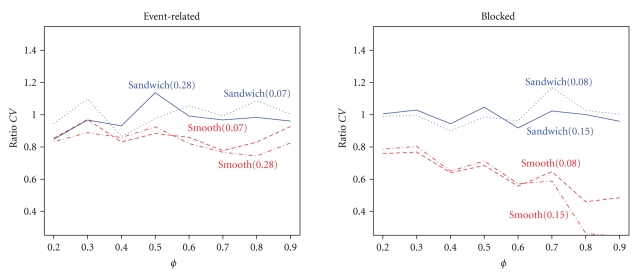
Ratios of estimated and true contrast variance for the event-related and blocked design when both the correlation structure and HRF model are incorrect. Two cuts of both the sandwich (blue) and smoothing approach (red) variance estimates are shown, at *δ* = 0.07 and 0.28 for event-related, and at *δ* = 0.08 and 0.15 for blocked design.

In addition to misspecification of the correlation structure the HRF model can be misspecified. To look at possible interactions with correlation strength, we varied both HRF misspecification and correlation strength. As can be seen in Figure 8, for the event-related design the sandwich is more accurate than the smoothing approach, which underestimates the contrast variance. But there is only a small effect of HRF misspecification for both the sandwich and smoothing approach. For the blocked design, on the other hand, the smoothing approach underestimates contrast variance greatly. Accordingly, the FPR of the smoothing approach in the event-related design is too low, around 2.5%. This is due to overcompensation of the degrees of freedom *f*_*S*_ in the smoothing approach. When there are no autocorrelations, *f*_*S*_ is high, and when there are autocorrelations, *f*_*S*_ is low. When the HRF is modeled incorrectly, *f*_*S*_ is too low so that the FPR is too low. In the blocked design the FPR behaves as expected for the smoothing approach: the contrast variance is underestimated which leads to overestimated FPR. The sandwich remains in both designs relatively stable around 5%. The power behaves as expected in this case (not shown): for the smoothing approach the power is similar to that in Figure 7 for the event-related design and higher for the blocked design. The power of the sandwich is similar to that of Figure 7.

## 4. Discussion

It has been repeatedly shown that the false positive rate in fMRI brain activity maps can be quite high if the assumptions of the method are violated (see, e.g., [4, 7]). Therefore, the robustness of the variance estimator of the GLM coefficients is an important issue. It has been shown here that the sandwich is unbiased and accordingly an exact *F*-test with the sandwich exists. Additionally, misspecifications in both autcorrelation and HRF model are accommodated by the sandwich for both event-related and blocked designs. In contrast, the smoothing approach is affected by both autocorrelation and HRF misspecification. Additionally, the smoothing approach requires a smoothing parameter which must be specified for each correlation structure to get accurate results. In contrast, the sandwich variance has two main advantages to the smoothing approach: (i) the sandwich adapts to local changes in correlation structure, whereas the smoothing approach does not, and (ii) no model or parameter needs to be determined for accurate results with the sandwich.

The potential of the application of the sandwich to real data is large. For example, we have applied the sandwich to real fMRI data in Weeda et al. [37]. In that paper we took a multivariate approach to model the GLM coefficients using Gaussian shaped functions. Using an incorrect shape function and incorrect autocorrelation assumptions, we showed that the contrast variance is still accurate of the sandwich. Using the sandwich we were able to find a plausible set of areas of brain activity in an auditory task.

Another area where the sandwich can be used is random effects analysis [38], which is our current work. The first level of a two-level random effects model requires only an OLS estimate of the coefficient of each subject and its sandwich. Then at the second level, the group effects are estimated with OLS again, and another sandwich is formed which is simply the sandwich of the first-level variance with the group design for all subjects.

## Figures and Tables

**Figure 9:**
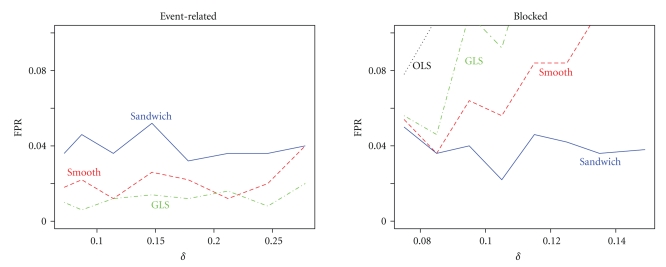
False positive rate as a function of relative difference *δ* for the event-related and blocked design when both the correlation structure and HRF model are incorrect. The correlation strength was *ϕ* = 0.9.

**Table 1:** The four methods of estimation and their corresponding variance.

Type	Mean	Variance
*W*	${\hat{\beta }}_{O}=(X^{\prime }X{)}^{-1}X^{\prime }\overset{̅}{Y}$	${\hat{V}}_{W}=\frac{1}{n}(X^{\prime }X{)}^{-1}X^{\prime }WX(X^{\prime }X{)}^{-1}$
OLS	${\hat{\beta }}_{O}=(X^{\prime }X{)}^{-1}X^{\prime }\overset{̅}{Y}$	${\hat{V}}_{O}={\hat{\sigma }}_{O}^{2}(X^{\prime }X{)}^{-1}$
GLS	${\hat{\beta }}_{G}=(X^{\prime }\hat{R}X{)}^{-1}X^{\prime }\hat{R}\overset{̅}{Y}$	${\hat{V}}_{G}={\hat{\sigma }}_{G}^{2}(X^{\prime }\hat{R}X{)}^{-1}$
*S*	${\hat{\beta }}_{S}=(X^{\prime }S^{\prime }SX{)}^{-1}X^{\prime }S^{\prime }S\overset{̅}{Y}$	${\hat{V}}_{S}={\hat{\sigma }}_{S}^{2}(X^{\prime }S^{\prime }SX{)}^{-1}X^{\prime }S^{\prime }S\hat{R}S^{\prime }SX(X^{\prime }S^{\prime }SX{)}^{-1}$

## Appendix

To prove the distributional result of the statistic *F*_*W*_ we assume three things: (i) the DGP as stated in Section 2, (ii) the working model of Section 2, and (iii) the noise is multivariate normal, that is, *F*(*e*) = *N*_*p*_(0, Σ). Then, to prove that *F*_*W*_ is central *F* distributed with degrees of freedom *q* and *n* − *q*, we need to show that (i) the variance $C{\hat{V}}_{W}C^{\prime }$ is Wishart distributed, (ii) $C\hat{\beta }$ and ${\hat{V}}_{W}$ are independent, and (iii) the degrees of freedom are *q* and *n* − 1 (see, e.g., [39, chapter 7 and 8]). (i) By Proposition 7.4 of [39] we have that if $\left(n-1){\hat{V}}_{W}\sim W_{k}(n-1,V\right)$, then $\left(n-1\right)C{\hat{V}}_{W}C^{\prime }\sim W_{q}(n-1,CVC^{\prime })$, where $V=\text{var}\{{\hat{\beta }}_{O}\}$. So, if ${\hat{V}}_{W}$ is Wishart distributed, we are done. Rewrite ${\hat{V}}_{W}$, such that if *U*_*j*_ = (*X*′*X*)^−1^ *X*′*r*_*j*_, then $n(n-1){\hat{V}}_{W}=\sum_{j=1}^{n}U_{j}U_{j}^{\prime }$. Now *U*_*j*_ is *N*_*k*_(0, (*n* − 1)*V*). This is seen by noting that *E*{*U*_*j*_} = (*X*′*X*)^−1^ *X*′*Q*_*X*_ *g*_*θ*_(*Z*) = 0 and var{*U*_*j*_} = ((*n* − 1)/*n*)(*X*′*X*)^−1^ *X*′Σ*X*(*X*′*X*)^−1^, because of the variance of the residuals. Then by definition $\left(n-1){\hat{V}}_{W}\sim W_{k}(n-1,V\right)$. For (ii), to show independence of $C{\hat{\beta }}_{O}$ and ${\hat{V}}_{W}$, it is sufficient to show independence of ${\hat{\beta }}_{O}$ and *U*_*j*_. Because the data are normal by assumption, the covariance of ${\hat{\beta }}_{O}$ and *U*_*j*_ needs to be zero to show independence. Since the covariance of ${\left({\overset{̅}{Y}}^{\prime },r_{j}^{\prime }\right)}^{\prime }$ is (1/*n*)*Q*_*X*_Σ, it then follows that $C{\hat{\beta }}_{O}$ and ${\hat{V}}_{W}$ are independent. To show (iii), that the degrees of freedom are *q* for the numerator and *n* − 1 for the denominator, proposition 8.2 of [39] is used. It implies that if $C{\hat{\beta }}_{O}-a\sim N_{q}(0,CVC^{\prime })$ and $\left(n-1\right)C{\hat{V}}_{W}C^{\prime }\sim W_{q}(n-1,CVC^{\prime })$, then *F*_*W*_ ~ *F*(*q*, *n* − *q*). The first part is true under *H*_0_ and from the variance of the OLS estimate ${\hat{\beta }}_{O}$, and the second part was shown in (i).
